# Restricted Mean Survival Time in Action to Control Cardiovascular Risk in Diabetes (ACCORD) Blood Pressure

**DOI:** 10.1093/geroni/igaa057.1588

**Published:** 2020-12-16

**Authors:** Sandra Shi, Lee-Jen Wei, Dae Kim

**Affiliations:** 1 Beth Israel Deaconess Medical Center, Boston, Massachusetts, United States; 2 Harvard School of Public Health, Boston, United States; 3 Hebrew SeniorLife, Harvard Medical School, Roslindale, Massachusetts, United States

## Abstract

Background: Restricted mean survival time (RMST) provides mean time lost or gained by an intervention. This may be a more intuitive way to understand treatment effect. Objective: To determine overall and subgroup treatment effects from blood pressure targets in older adults with diabetes. Methods: We analyzed the Action to Control Cardiovascular Risk in Diabetes (ACCORD) trial data. Our outcome was cardiovascular disease free survival. We measured 5-year RMST (days) between standard and intensive blood pressure control, and compared the difference by age (≥70 or <70 years old) and glycemic control (standard vs. intensive). Results: Over 5 years, those with intensive treatment lived, on average, 1716 days compared to 1714 days with standard treatment, with a RMST difference of 1.3 days (95% confidence interval, -22.1, 12.4). Among adults ≥70 years old, compared to standard treatment, intensive treatment resulted in an additional 13.6 (95% CI, -43.2, 70.3) days, where the difference was -0.5 (-20.8, 19.7) days for those aged <70 years old (p-for-interaction=0.673). Compared to standard treatment, intensive treatment resulted in 28.1 (0.4, 55.9) more days for those assigned to standard glycemic control, but it appeared to result in 25.2 fewer days (-52.3, 1.9) for those assigned to intensive glycemic control (p-for-interaction=0.007). Discussion: The benefit of intensive treatment over standard treatment varies by age and glycemic control. RMST difference may allow for more intuitive and personalized weighing of benefits and risks of intensive blood pressure control.

